# Anti-Inflammatory Effect of Baicalein on Polyinosinic–Polycytidylic Acid-Induced RAW 264.7 Mouse Macrophages

**DOI:** 10.3390/v10050224

**Published:** 2018-04-26

**Authors:** Young-Jin Kim, Hyun-Ju Kim, Ji Young Lee, Do-Hoon Kim, Mi Suk Kang, Wansu Park

**Affiliations:** College of Korean Medicine, Gachon University, Seong-Nam 13120, Korea; godsentry@naver.com (Y.-J.K.); eternity0304@daum.net (H.-J.K.); oxygen1119@naver.com (J.Y.L.); chulian@gachon.ac.kr (D.-H.K.); cyberdoc@gachon.ac.kr (M.S.K.)

**Keywords:** baicalein, dsRNA, inflammation, macrophages, nitric oxide, cytokine, calcium, STAT, CHOP

## Abstract

Baicalein (3,3′,4′,5,6-pentahydroxyflavone) is a well-known antioxidant found in many plants, such as in the roots of *Scutellaria baicalensis*. In this study, we evaluate the inhibitory effect of baicalein on the inflammatory cascade in RAW 264.7 mouse macrophages induced by viral-like material. Experimental assays used in this study included Griess reagent assay for nitric oxide (NO) production, Fluo-4 assay for intracellular calcium release, multiplex cytokine assay, and quantitative real time RT-PCR assay. To induce inflammation, RAW 264.7 cells were treated with polyinosinic–polycytidylic acid (poly I:C), a synthetic analog of double-stranded RNA (dsRNA). Baicalein at concentrations up to 100 μM significantly inhibited the production of NO, IL-1α, IL-6, G-CSF, GM-CSF, VEGF, MCP-1, IP-10, LIX, and RANTES as well as calcium release in RAW 264.7 cells induced by poly I:C (50 µg/mL) (all *p* < 0.05). Baicalein at concentrations up to 50 μM also significantly inhibited mRNA expression of *STAT1*, *STAT3*, *CHOP*, and *Fas* in poly I:C-induced RAW 264.7 cells (*p* < 0.05). In conclusion, baicalein has anti-inflammatory effect in double-stranded RNA (dsRNA)-induced macrophages by inhibiting NO, cytokines, chemokines, and growth factors via the endoplasmic reticulum stress–CHOP/STAT pathway.

## 1. Introduction

**Figure 1:**
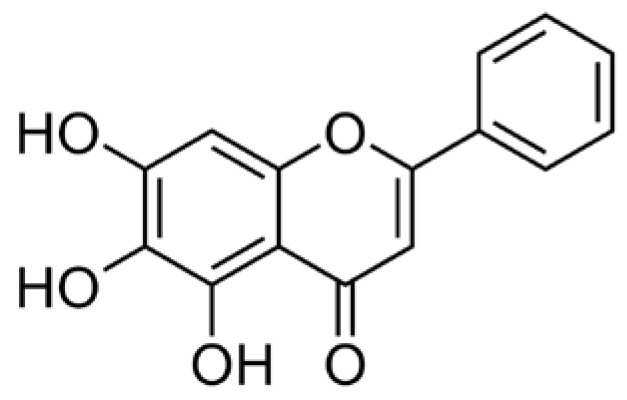
Structural formula of the baicalein flavonoid.

Baicalein (3,3′,4′,5,6-pentahydroxyflavone; Figure 1) is a well-known antioxidant found in many plants including roots of Baikal skullcap (*Scutellaria baicalensis*), American skullcap (*Scutellaria lateriflora*), and Indian trumpet flower (*Oroxylum indicum*). In 2013, Fan et al. reported that baicalein, a major constituent of *Scutellaria radix*, exerted anti-inflammatory activities in lipopolysaccharide (LPS)-induced RAW 264.7 cells by inhibiting mRNA expression of nitric oxide synthase (iNOS) and cyclooxygenase-2 (COX-2) [1] and, in 2017, Gasparrini et al. reported the health benefits of strawberries against inflammatory and oxidative stress in LPS-stimulated RAW 264.7 [2]. Lee et al. also suggested that baicalein can inhibit hyperpermeability, expression of cell adhesion molecules (CAMs), and adhesion and migration of leukocytes [3].

We have previously evaluated immuno-inflammatory activities of various natural products with respect to macrophages [4,5,6,7,8,9,10,11]. Additionally, it was demonstrated that the intracellular signaling pathway triggered by Toll-like receptor 3 can be modulated by some natural products [8,9,10]. However, the inhibitory effect of baicalein on macrophages induced by viral infection has not yet been reported.

Therefore, the objective of this study was to evaluate the in vitro inhibitory effect of baicalein on inflammatory cascade in RAW 264.7 cells induced by viral-like material such as double-stranded RNA (dsRNA) known to be presented in viral infection. Polyinosinic–polycytidylic acid (poly I:C), a synthetic analog of dsRNA, was used to induce RAW 264.7 cells in this study. We have previously reported that wogonin, oroxylin A, and quercetin exert anti-inflammatory effects on RAW 264.7 cells induced by poly I:C [8,9,10]. Like our previous reports, multiplex cytokine assay was also used to verify details of cytokines meaningful for evaluating the anti-inflammatory activity of baicalein against viral infection.

Our experimental data presented that baicalein significantly inhibited production of nitric oxide (NO), interleukin (IL)-1α, IL-6, vascular endothelial growth factor (VEGF), granulocyte colony-stimulating factor (G-CSF), granulocyte macrophage colony-stimulating factor (GM-CSF), monocyte chemotactic protein (MCP)-1, interferon-inducible protein (IP)-10, lipopolysaccharide-induced CXC chemokine (LIX; CXCL5), and chemokine ligand 5 (CCL5; RANTES) as well as calcium release in RAW 264.7 cells induced by poly I:C. In addition, baicalein significantly inhibited mRNA expression levels of signal transducer and activated transcription 1 (*STAT1*), *STAT3*, C/EBP-homologous protein (*CHOP*; Ddit3), and first apoptosis signal receptor (*Fas*) in RAW 264.7 cells induced by poly I:C.

## 2. Materials and Methods

### 2.1. Materials

DMEM was purchased from Gibco BRL (Grand Island, NY, USA). Baicalein was obtained from Sigma-Aldrich (St. Louis, MO, USA) and used for experiments without the additional purification. Baicalein at the concentration of 20 mM was prepared in DMSO and then further solubilized in aqueous solutions. All other chemicals for the cell culture were obtained from Merck Millipore (Darmstadt, Germany).

### 2.2. Cell Culture

RAW 264.7 were obtained from Korea Cell Line Bank (Seoul, Korea). RAW 264.7 (passage number 6) were cultured with DMEM according to the protocol of previous study [4]. Before experimental assays, RAW 264.7 were washed with phosphate buffer saline. In this study, with the modified MTT assay [4], baicalein up to a concentration of 50 μM restored the cell viability in poly I:C-induced RAW 264.7.

### 2.3. NO Assay

It is well known that Griess reagent is useful to detect the levels of nitrite in aqueous solutions. After 24-h culture in 96-well plates, NO levels in each well containing 10,000 cells were identified using the Griess reagent (Merck Millipore) according the protocol of our previous study [4,5,6]. Briefly, after incubating the cells with poly I:C and/or baicalein for 24 h, 100 µL of supernatant from each well were mixed with 100 µL Griess reagent in a 96-well plate. After 15 min of incubation at room temperature, optical density was determined at 540 nm with a microplate reader (Bio-Rad, Hercules, CA, USA). NO assay showed that poly I:C (50 µg/mL) induces RAW 264.7 to produce NO significantly. 

### 2.4. Intracellular Calcium Assay

Fluo-4 AM is a well-known fluorescent Ca^2+^ indicator used for the in-cell measurement of calcium signaling. After 18 h of treatment in 96-well plates, the intracellular calcium signaling from each well containing 100,000 cells was identified using Fluo-4 NW Calcium Assay Kits (Thermo Fisher Scientific, Waltham, MA, USA) according the protocol of our previous study [8,9,10]. Specifically, after incubating the cells with poly I:C and/or baicalein for 18 h at 37 °C, the medium was removed and the cells were subsequently incubated with 100 µL of the Fluo-4 dye loading solution for 30 min at 37 °C. After the incubation, fluorescence intensity in each well was determined spectrofluorometrically (Dynex, West Sussex, UK) with excitation and emission filters of 485 nm and 535 nm, respectively.

### 2.5. Multiplex Bead-Based Cytokine Assay

After RAW 264.7 cells were seeded in wells of a 96-well plate, poly I:C and/or baicalein were added to the culture medium, and incubation was continued for 24 h at 37 °C. The supernatant was collected from each well containing 10,000 cells, and cytokines were measured using a Luminex assay based on xMAP technology. This assay was performed with Milliplex kits (Millipore, Billerica, MA, USA) and Bio-Plex 200 suspension array system (Bio-Rad) as described previously [8,9,10,11]. The production of the following cytokines was analyzed: IL-1α, IL-6, G-CSF, GM-CSF, VEGF, IP-10, LIX, MCP-1, RANTES, and tumor necrosis factor-alpha (TNF-α). Indomethacin was used as a positive control. Standard curves for each cytokine were generated using the kit-supplied reference cytokine samples.

### 2.6. RNA Isolation and Real Time RT-PCR Analysis

In six-well plates, 300,000 RAW 264.7 cells were incubated in each well with or without baicalein in poly I:C for 18 h. At the end of the 18-h incubation with poly I:C and/or baicalein, cells were lysed and total RNA was isolated using NucleoSpin RNA kit (Macherey-Nagel, Duren, Germany), and then reverse transcribed into cDNA with iScript cDNA Synthesis kit (Bio-Rad). RNA quantity and quality were confirmed using the Experion RNA StdSens Analysis kit (Bio-Rad) and Experion Automatic Electrophoresis System (Bio-Rad). The cDNA amplification was carried out to detect the target genes (*STAT1*, *STAT3*, *CHOP*, *Fas*, and *TBP*) using iQ SYBR Green Supermix (Bio-Rad) according to the previous study [9]. The *TBP* was used as a reference. Primers used are listed in Table 1. Berberine was used as a **positive control.**

### 2.7. Statistical Analysis

Experimental results are presented as mean ± SD. Experiments were done more than three times. Data were analyzed by one-way analysis of variance test followed by Tukey’s multiple comparison test of SPSS software (ver. 11; SPSS Inc., Chicago, IL, USA). A *p*-value <0.05 was considered statistically significant.

## 3. Results

### 3.1. Effect of Baicalein on NO Production and Intracellular Calcium Release

**Figure 2:**
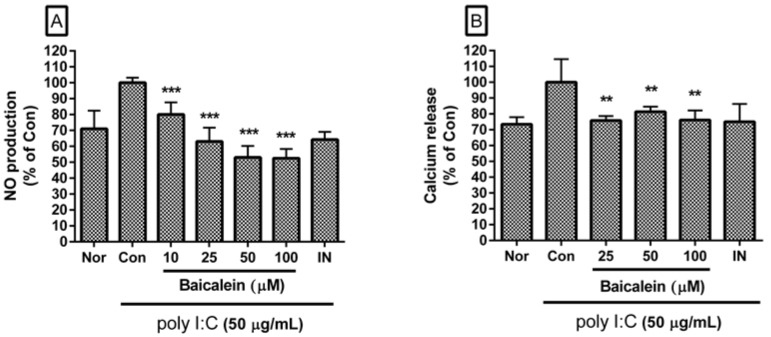
Effect of baicalein on NO production (**A**) and calcium release (**B**) by RAW 264.7 cells induced by poly I:C. The normal group (Nor) was treated with media only. The control group (Con) was treated with poly I:C (50 µg/mL) alone. IN: indomethacin (0.5 µM). Values are mean ± SD. ** *p* < 0.01 vs. Con; *** *p* < 0.001. poly I:C: polyinosinic–polycytidylic acid.

Baicalein significantly inhibited excessive production of NO in poly I:C-induced RAW 264.7 cells (Figure 2A). Percentages of NO production in poly I:C-induced RAW 264.7 cells incubated with baicalein at concentrations of 10, 25, 50, and 100 µM for 24 h were 80.02 ± 7.64%, 62.0 ± 8.72%, 53.01 ± 7.28%, and 52.6 ± 5.74% of the control value (IC_50_ value of 63.59 µM), respectively.

Baicalein significantly inhibited calcium release in poly I:C-induced RAW 264.7 cells (Figure 2B). Percentages of calcium release in poly I:C-induced RAW 264.7 cells incubated with baicalein at concentrations of 25, 50, and 100 µM for 18 h were 75.82 ± 2.79%, 81.32 ± 3.21%, and 76.1 ± 6.01% of the control value, respectively.

### 3.2. Effect of Baicalein on Cytokine Production

**Figure 3:**
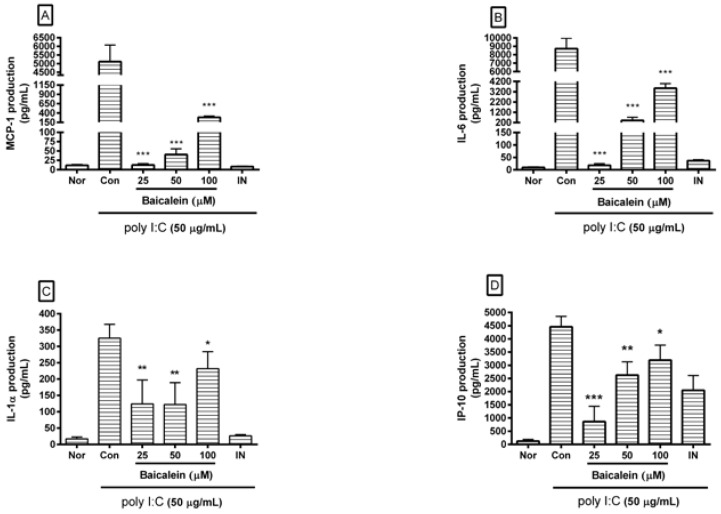

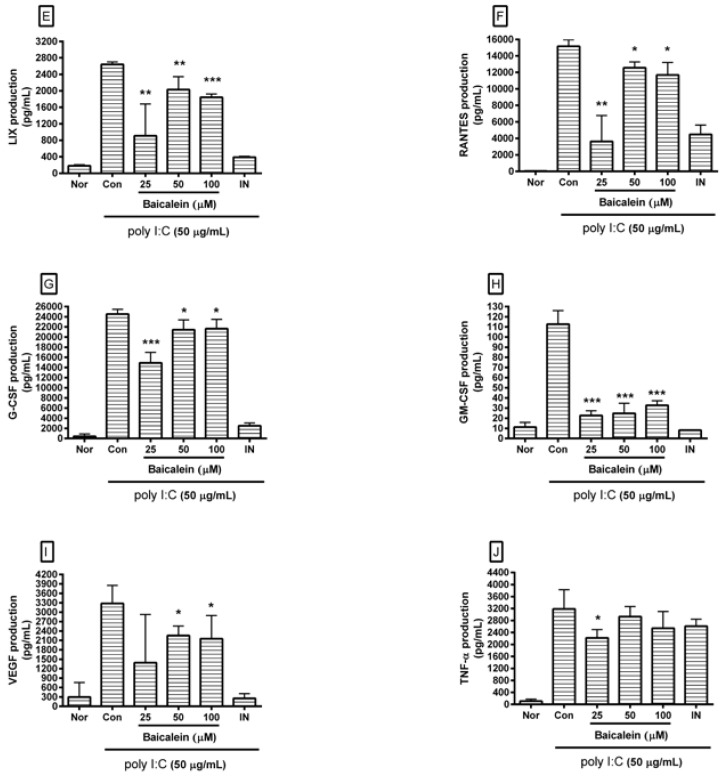
Effect of baicalein on production of cytokines such as monocyte chemotactic protein (MCP)-1 (**A**); interleukin (IL)-6 (**B**); IL-1α (**C**); interferon-inducible protein (IP)-10 (**D**); lipopolysaccharide-induced CXC chemokine (LIX; CXCL5) (**E**); Chemokine ligand 5 (CCL5; RANTES) (**F**); granulocyte colony-stimulating factor (G-CSF) (**G**); granulocyte macrophage colony-stimulating factor (GM-CSF) (**H**); vascular endothelial growth factor (VEGF) (**I**); and tumor necrosis factor-alpha (TNF-α) (**J**) by RAW 264.7 cells induced by poly I:C. Nor was treated with media only. Con was treated with poly I:C (50 µg/mL) alone. IN: indomethacin (0.5 µM). Values are mean ± SD. * *p* < 0.05 vs. Con; ** *p* < 0.01; *** *p* < 0.001.

After incubating cells with poly I:C and/or baicalein for 24 h, cytokines released from cells were measured in cell culture supernatants using Luminex assay based on xMAP technology. Results showed that baicalein significantly reduced excess production of IL-1α, IL-6, G-CSF, GM-CSF, VEGF, MCP-1, IP-10, LIX, and RANTES in RAW 264.7 cells induced by poly I:C (Figure 3).

### 3.3. Effect of Baicalein on mRNA Expression of STAT1, STAT3, CHOP and Fas

**Figure 4:**
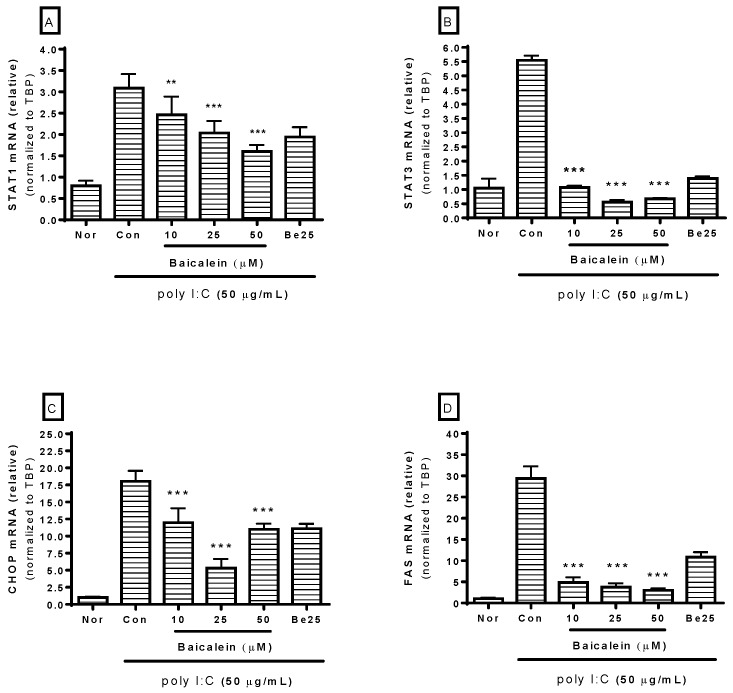
Effect of baicalein on mRNA expressions of *STAT1* (**A**), *STAT3* (**B**), *CHOP* (**C**), and *Fas* (**D**) in RAW 264.7 cells induced by poly I:C. After 18 h of treatment, mRNA expressions of *STAT1*, *STAT3*, *CHOP*, and *Fas* were measured by real-time reverse-transcription polymerase chain reaction assay. *STAT1*, *STAT3*, *CHOP*, and *Fas* mRNA levels were normalized to *TBP* mRNA. Normal group (Nor) was treated with media only. Control group (Con) was treated with poly I:C (50 µg/mL) alone. Be25: berberine (25 µM). Values are mean ± standard deviation of three independent experiments. ** *p* < 0.01 vs. Con; *** *p* < 0.001.

Because STAT, CHOP, and Fas are involved in macrophage activation, mRNA expression of *STAT1*, *STAT3*, *CHOP*, and *Fas* in RAW 264.7 cells incubated with poly I:C was investigated in this study. After incubating cells with poly I:C and/or baicalein for 18 h, real-time reverse-transcription polymerase chain reaction (RT-PCR) was performed to determine mRNA expressions of *STAT1* (GenBank: NM_009283), *STAT3* (GenBank: NM_213659), *CHOP* (GenBank: NM_007837), and *Fas* (GenBank: NM_007987). The *TBP* gene (GenBank: NM_013684) was used for RNA normalization. Results showed that baicalein significantly inhibited mRNA expressions of *STAT1*, *STAT3*, *CHOP*, and *Fas* in RAW 264.7 cells induced by poly I:C at concentrations of 10, 25, and 50 µM (Figure 4).

## 4. Discussion

In recent years, some viral infections such as MERS, Ebola and Zika have caused problems in public health in developing nations and developed nations. Due to the high number of variable and/or mutagenic pathogenic viruses, it seems to be reasonable to search for new vaccine and novel anti-inflammatory material against these rising viral pathogens.

Many studies have reported that natural products possess meaningful effects on inflammation caused by bacterial endotoxin as well as other infectious pathogens such as viruses and fungi. In 2012, Hong et al. reported that cinnamon water extract could decrease serum levels of TNF-α and IL-6 in LPS-induced mice [12]. Cinnamon water extract treatment in vitro decreased mRNA expression of TNF-α via modulating inhibitor protein-κBα (IκBα) degradation and p38 mitogen-activated protein kinase (p38), extracellular signal-regulated kinases 1/2 (ERK1/2), and c-Jun N-terminal protein kinase (JNK) activation [12]. In 2005, Duarte et al. reported that essential oils from 13 plants showed anti-candida activity, including *Aloysia triphylla*, *Anthemis nobilis*, *Cymbopogon martini*, *Cymbopogon winterianus*, *Cyperus articulatus*, *Cyperus rotundus*, *Lippia alba*, *Mentha arvensis*, *Mikania glomerata*, *Mentha piperita*, *Stachys byzantina*, and *Solidago chilensis* [13]. In 2017, Li et al. reported that polysaccharide extract from *Radix isatidis* exerted potent anti-influenza A virus activity against human seasonal influenza viruses (subtypes H1N1 and H3N2) and avian influenza viruses (subtypes H6N2 and H9N2) in vitro [14]. Such polysaccharides also significantly reduced the expression of IL-6 and chemokines (IP-10, MIG and CCL-5) stimulated by the H1N1 virus [14]. 

Among natural products, the root of *Scutellaria Baicalensis* and its flavonoids have attracted attention from researchers due to their anti-inflammatory activities. In 2009, Li-Weber reported that the major compounds of *Scutellaria Baicalensis* (baicalein, baicalin, and wogonin) were cytotoxic to various human tumor cell lines in vitro [15]. They also inhibited tumor growth in vivo [15]. Li et al. also reported that flavonoid baicalin exhibits anti-inflammatory activity by binding to chemokines [16]. However, the inhibitory effect of baicalein on macrophages induced by viral infection has not been reported until now.

During viral infection in the human body, it is well known that double-stranded RNA are produced through viral replication. These pathogen-originated alien materials might be hazardous enough to provoke excessive inflammatory process in the host [17]. In the present study, a synthetic analog of dsRNA (poly I:C) was used to generate the in vitro model of viral inflammation. In short, viral analog poly I:C was used to induce RAW 264.7 cells so that various kinds of inflammatory mediators such as NO, cytokines, chemokines, growth factors, and prostaglandins etc. were discharged from activated macrophages. Consequentially, adaptable and nontoxic anti-inflammatory materials have been tested to determine whether they can modulate hyper-production of inflammatory mediators that can affect homeostasis in human body. These inflammatory mediators can be even mortal to human life. 

In 2001, Alexopoulou et al. reported that mammalian Toll-like receptor 3 could recognize dsRNA and gradually induce cytokine production via mitogen-activated protein (MAP) kinase activation [17]. Our data revealed that baicalein significantly inhibited the production of NO, cytokines (IL-1α and IL-6), chemokines (MCP-1, IP-10, and LIX), and growth factors (G-CSF, GM-CSF, and VEGF), as well as calcium release, which was massive in poly I:C-induced RAW 264.7 cells. These results indicate that one of the possible modulatory effects on viral inflammation regulated by baicalein involves the calcium signal pathway, which is in accordance with our previous reports on the inhibitory effects of wogonin [8], quercetin [9], and oroxylin A [10] on an in vitro viral inflammatory model. It is necessary to clarify the effect of baicalein on hyper-production of cytokines (cytokine storm) during viral infectious diseases.

It is well known that the influx of calcium in inflammatory reaction can initiate a cascade that finally evokes NO release in inflammatory reaction [18]. Intracellular calcium level is increased during infectious inflammation via calcium influx by the transient receptor potential melastatin 4 (TRPM4) channel [19] and release of endoplasmic reticulum (ER) calcium stored in the cytoplasm through the ER stress pathway in activated macrophages [20]. In 2007, Stout et al. reported that STAT1 activation could elicit ER stress that may cause mucous cell hyperplasia in chronic asthma [21]. Additionally, Timmins et al. in 2009 reported that calcium increase in macrophages might induce Fas expression [22]. In 2011, Lim et al. reported that CHOP expression was increased via ER stress in macrophages infected with *Mycobacterium tuberculosis* [23]. In this study, baicalein significantly inhibited expressions of *CHOP*, *STAT1*, *STAT3*, and *Fas* mRNAs in RAW 264.7 cells induced by poly I:C. This means that baicalein may be able to alleviate ER stress induced by dsRNA via the CHOP/STAT pathway.

Further study is needed to evaluate why higher concentrations of baicalein resulted in less inhibition of cytokine expression in this study.

## 5. Conclusions

Baicalein at concentrations up to 100 μM significantly inhibited the production of NO, IL-1α, IL-6, G-CSF, GM-CSF, VEGF, MCP-1, IP-10, LIX, and RANTES, as well as calcium release in poly I:C-induced RAW 264.7 cells (all *p* < 0.05). Baicalein at concentrations up to 50 μM also significantly inhibited mRNA expression levels of *STAT1*, *STAT3*, *CHOP*, and *Fas* in poly I:C-induced RAW 264.7 cells (all *p* < 0.05). These results indicate that baicalein has anti-inflammatory effects on dsRNA-induced macrophages by inhibiting production of NO, cytokines, chemokines, and growth factors via the endoplasmic reticulum stress–CHOP/STAT pathway.

## Figures and Tables

**Table 1:** Primers used for RT-PCR analysis.

Name	Forward Primer (5′–3′)	Reverse Primer (5′–3′)
*STAT1*	TGAGATGTCCCGGATAGTGG	CGCCAGAGAGAAATTCGTGT-3
*STAT3*	GTCTGCAGAGTTCAAGCACCT	TCCTCAGTCACGATCAAGGAG
*CHOP*	CGCTGTTTTCCCTTGCTG	TCCTCATACCAGGCTTCCA
*Fas*	CGCTGTTTTCCCTTGCTG	CCTTGAGTATGAACTCTTAACTGTGAG
*TBP*	GGGGAGCTGTGATGTGAAGT	CCAGGAAATAATTCTGGCTCA
